# Development of a prognostic nomogram and risk stratification system for upper thoracic esophageal squamous cell carcinoma

**DOI:** 10.3389/fonc.2023.1059539

**Published:** 2023-04-12

**Authors:** Yu Lin, Binglin Zheng, Junqiang Chen, Qiuyuan Huang, Yuling Ye, Yong Yang, Yuanmei Chen, Bijuan Chen, Mengxing You, Qifeng Wang, Yuanji Xu

**Affiliations:** ^1^Department of Radiation Oncology, Clinical Oncology School of Fujian Medical University, Fujian Cancer Hospital, Fuzhou, China; ^2^Department of Radiation Oncology, Fujian Medical University Union Hospital, Fuzhou, China; ^3^Department of Thoracic Surgery, Clinical Oncology School of Fujian Medical University, Fujian Cancer Hospital, Fuzhou, China; ^4^Department of Medical Oncology, The First Hosptial of Putian, Fujian Medical University Teaching Hospital, Putian, China; ^5^Department of Radiation Oncology, Sichuan Cancer Hospital Institute, Sichuan Cancer Center, School of Medicine, University of Electronic Science and Technology of China, Chengdu, China

**Keywords:** upper thoracic ESCC, nomogram model, prognosis, risk stratification, AJCC stage

## Abstract

**Background:**

The study aimed to develop a nomogram model to predict overall survival (OS) and construct a risk stratification system of upper thoracic esophageal squamous cell carcinoma (ESCC).

**Methods:**

Newly diagnosed 568 patients with upper ESCC at Fujian Medical University Cancer Hospital were taken as a training cohort, and additional 155 patients with upper ESCC from Sichuan Cancer Hospital Institute were used as a validation cohort. A nomogram was established using Cox proportional hazard regression to identify prognostic factors for OS. The predictive power of nomogram model was evaluated by using 4 indices: concordance statistics (C-index), time-dependent ROC (ROCt) curve, net reclassification index (NRI) and integrated discrimination improvement (IDI).

**Results:**

In this study, multivariate analysis revealed that gender, clinical T stage, clinical N stage and primary gross tumor volume were independent prognostic factors for OS in the training cohort. The nomogram based on these factors presented favorable prognostic efficacy in the both training and validation cohorts, with concordance statistics (C-index) of 0.622, 0.713, and area under the curve (AUC) value of 0.709, 0.739, respectively, which appeared superior to those of the American Joint Committee on Cancer (AJCC) staging system. Additionally, net reclassification index (NRI) and integrated discrimination improvement (IDI) of the nomogram presented better discrimination ability to predict survival than those of AJCC staging. Furthermore, decision curve analysis (DCA) of the nomogram exhibited greater clinical performance than that of AJCC staging. Finally, the nomogram fairly distinguished the OS rates among low, moderate, and high risk groups, whereas the OS curves of clinical stage could not be well separated among clinical AJCC stage.

**Conclusion:**

We built an effective nomogram model for predicting OS of upper ESCC, which may improve clinicians’ abilities to predict individualized survival and facilitate to further stratify the management of patients at risk.

## 1 Introduction

Esophageal cancer (EC) is a common digestive tract tumor ranking seventh in incidence and sixth in mortality worldwide. For example, in 2020, nearly 50% of EC cases and deaths worldwide occurred in China (1). Esophageal squamous cell carcinoma (ESCC) is the most common pathological type in China, accounting for 90% of EC (2). According to the guidelines of the National Comprehensive Cancer Network (NCCN), surgery is the first choice for treatment of ESCC (3). Nevertheless, there is less opportunity for surgery to treat upper thoracic ESCC because of its unique anatomical position and high risk of local invasion. The survival rate of upper thoracic ESCC remains poor, with a 5-year overall survival (OS) rate of 33% (4). Therefore, it is necessary to develop an effective clinical prognostic model to determine the prognosis of patients with upper thoracic ESCC and guide the selection of individualized treatment.

Several studies focus on an accurate prognostic model for patients with EC, using a nomogram (5–9). For patients with thoracic ESCC after radical esophagectomy, nomograms based on clinical prognostic factors or immunoscores effectively predict OS, which appears superior to the American Joint Committee on Cancer (AJCC) staging system (5, 6). Furthermore, for patients with EC treated with neoadjuvant chemoradiotherapy plus surgery, the prognostic nomograms accurately predict OS rates of internal and external validation cohorts (7, 8). Finally, for patients with ESCC undergoing definitive chemoradiotherapy, pretreatment prognostic factors including hematological indicators, concurrent chemoradiotherapy, radiotherapy, and clinical stage are incorporated into the nomogram, which achieves better accuracy to predict survival than the AJCC staging system and may be more practical for decision-making in individualized therapy (9).

Upper thoracic ESCC is characterized by its much lower morbidity and higher mortality than other locations of ESCC, which suggests that significant clinical differences may exist (4, 10). However, to our knowledge, no useful model effectively predicts the prognosis of patients with upper thoracic ESCC. Although the new AJCC staging system is widely used for predicting outcomes of patients with upper ESCC in clinical practice, we identified the primary gross tumor volume (GTVp) as an independent prognostic factor in our previous study (11). Consequently, the current AJCC staging may not be adequate for precisely predicting prognosis of upper ESCC. Moreover, the majority of previous nomogram models employ common parameters including concordance statistics (C-index) and areas under curve (AUC) (12, 13). Moreover, novel indicators such as the net reclassification index (NRI) and integrated discrimination improvement (IDI) are employed to evaluate the prognostic discrimination ability of the nomogram model (14, 15). Taken together, these findings illuminate the urgent requirement for a nomogram model that employs impactful prognostic evaluation parameters for upper ESCC.

The aim of the present study was to establish an effective nomogram model and develop a risk stratification system for upper thoracic ESCC. Hence, this study provides a clinical reference to predict prognosis and guide treatment of patients with upper ESCC.

## 2 Methods

### 2.1 Patients and pretreatment assessment

This study was approved by the Institutional Review Board of Fujian Medical University Cancer Hospital (No. SQ2020-063-01). The training cohort comprised 568 consecutive patients initially diagnosed with ESCC at Fujian Medical University Cancer Hospital from February 2004 to December 2016. The inclusion criteria for patient enrollment were as follows: no previous therapy, confirmed diagnosis of ESCC histologically or cytologically, clinical stage T1-4aN0-3M0, tumor located within the upper thoracic region (as defined by the NCCN guidelines) (3), and Eastern Cooperative Oncology Group (ECOG) performance score ≤3. Pretreatment assessment of all patients was performed according to our institutional protocol (16). The pretreatment evaluation for all patients included a neck and chest CT scan, abdominal ultrasonography or abdominal CT scan, and a whole-body bone single-photon emission computed tomography scan to rule out distant metastases. Additional tests and studies, such as positron emission tomography, were performed at the discretion of the treating physician. The GTVp was contoured by two experienced thoracic radiotherapists expert in interpreting chest computed tomography images before treatment, as described in our previous study (11). All patients were staged using the criteria of the eighth edition of the Union for International Cancer Control/American Joint Committee on Cancer staging criteria. Patients with secondary malignancies, recurrent disease, or other primary cancers located in the cervical, middle, or lower third of the thoracic esophagus were excluded. The other independent cohort of 155 patients, which was identified in the records of Sichuan Cancer Hospital by the same criteria between January 2011 and December 2013, served as a validation cohort.

### 2.2 Treatment and follow-up

Surgery including radical resection of the local tumor and regional lymph nodes was performed according to the procedures described in our previously published research (13). Patients underwent radiotherapy using three-dimensional conformal radiotherapy or intensity-modulated radiotherapy. The median irradiation doses delivered to patients using definitive radiotherapy, preoperative radiation, and postoperative radiation were 61.5 Gy (range, 50.0–67.2), 40.0 Gy (range, 36.0–50.0), and 50.0 Gy (range, 40.0–63.0), respectively. Details of radiotherapy planning and dose prescription were previously described (16–19). Platinum-based combination chemotherapy was applied in the present study.

Follow-up was performed every 3 months for the first 2 years, every 6 months for the next 3 years, and annually thereafter. Our clinical endpoint was OS, which was calculated as the interval from the date of diagnosis to the date of death or last follow-up. All patients were followed up until December 2019. The median follow-up time was 41.5 months.

### 2.3 Statistical analyses

The analyses of data were performed by SPSS version 24.0 and R version 4.0.3. OS rates estimated using the Kaplan–Meier method were compared using the log-rank test. Univariate and multivariate analyses were used to identify independent prognostic factors for OS using Cox proportional hazards regression, and *P <*0.05 was considered statistically significant. The hazard ratio (HR) with 95% confidence level (CI) was estimated as well. Accordingly, a nomogram model was built based on such significant prognostic factors for predicting 1-, 3-, and 5-year OS. The nomogram was subjected to bootstrapping validation (1000 bootstrap resamples) to calculate a relatively corrected C-index. A calibration curve was applied to assess the nomogram, and the AUC was used to quantify discriminative performance.

The accuracy of the nomogram model was evaluated using the calibration plot to compare the nomogram prediction with observed Kaplan–Meier estimates of the survival probability. We calculated NRI and IDI, comparing the nomogram model vs the AJCC staging system, which indicated improvement in predictive performance (20, 21). The nomogram established from the training cohort was tested using the validation cohort for testing prevalence. The model’s clinical utility was evaluated through decision curve analysis (DCA) based on net benefits that were calculated at a series of threshold probabilities (22, 23). Finally, according to the survival scores, the training cohort was categorized into low, moderate, and high-risk subgroups using X-tile software (24).

## 3 Results

### 3.1 Patients’ baseline characteristics and survival

Clinical characteristics of patients in the training and validation cohorts shown in **Table 1** include sex, age, lymph node metastasis (LNM), tumor length, GTVp, clinical T (cT) stage, clinical N (cN) stage, clinical TNM (cTNM) stage, and treatment. The median age was 60 years. The optimal cutoff of GTVp was defined as 30cm3 by our previous study (18). In addition, upper thoracic ESCCs were more frequent in males than females in both cohorts. With regard to staging, the majority of patients were staged as T2-3 and/or N0-1, and >50% of patients had lymph node metastasis. For the training and validation cohorts, 319 of 568 patients and 72 of 155 patients died, respectively. The 5-year OS rates of the training and validation cohort were 44.6% and 51.3%, respectively.

**Table 1:** Clinical characteristics of patients with upper thoracic esophageal squamous carcinoma in two cohorts.

	Training cohort (n=568)	Validation cohort (n=155)
Gender		
Male	391 (68.8)	123 (79.4)
Female	177 (31.2)	32 (20.6)
Age (years)		
< 60	276 (48.6)	61 (39.4)
≥ 60	292 (51.4)	94 (60.6)
LNM		
No	252 (44.4)	68 (43.9)
Yes	316 (55.6)	87 (56.1)
Tumor length (cm)		
≤ 5	363 (63.9)	109 (70.3)
> 5	205 (36.1)	46 (29.7)
GTVp (cm3)		
< 30	396 (69.7)	121 (78.1)
≥ 30	172 (30.3)	34 (21.9)
Clinical T stage		
T1	15 (2.6)	11 (7.1)
T2	112 (19.7)	23 (14.8)
T3	247 (43.5)	105 (67.7)
T4	194 (34.2)	16 (10.3)
Clinical N stage		
N0	285 (50.2)	68 (43.9)
N1	185 (32.6)	49 (31.6)
N2	88 (15.5)	22 (14.2)
N3	10 (1.8)	16 (10.3)
8^th^ AJCC stage		
I	10 (1.8)	11 (7.1)
II	251 (44.2)	68 (43.9)
III	117 (20.6)	46 (29.7)
IV	190 (33.5)	30 (19.4)
Treatment		
Surgery	238 (41.9)	70 (45.2)
CRT	216 (38.0)	0
Surgery+CRT	114 (20.1)	85 (54.8)

LNM, lymph node metastasis; GTVp, primary gross tumor volume; AJCC, American Joint Committee on Cancer.

### 3.2 Nomogram model construction and validation

**Figure 1:**
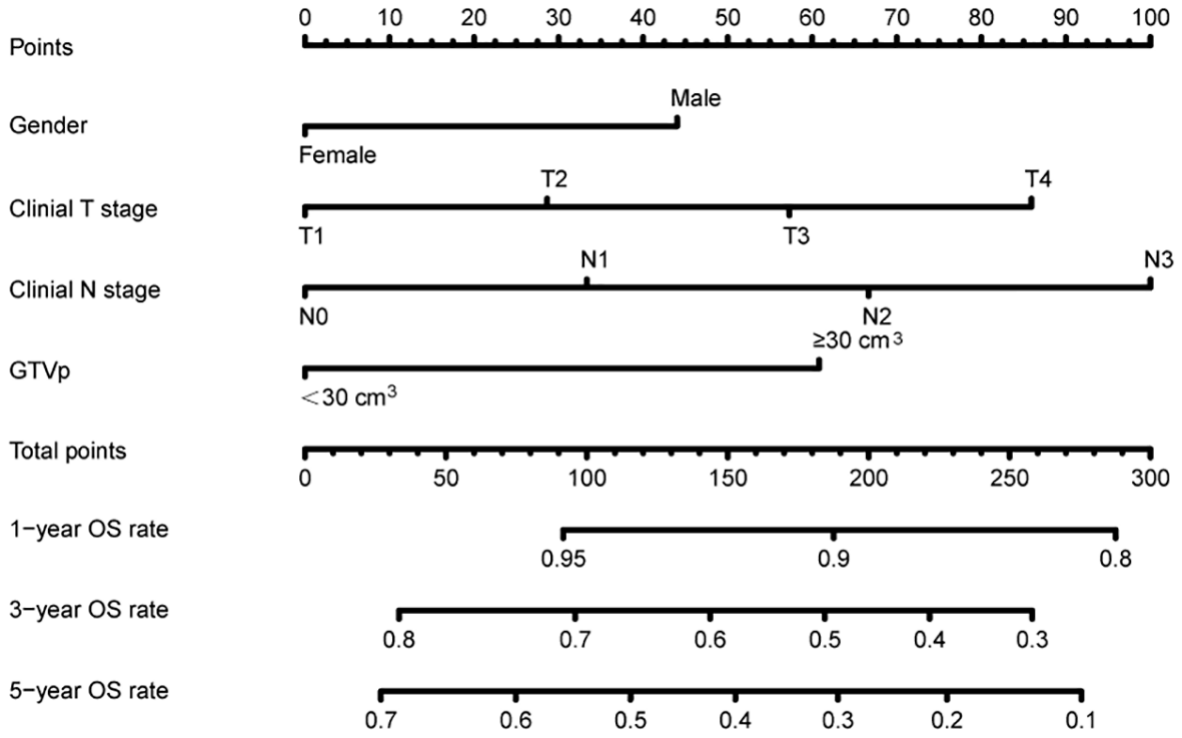
Nomogram predicting overall survival based on prognostic factors developed from the training cohort.

For the training cohort, univariate analysis revealed that sex, LNM, tumor length, GTVp, T stage, N stage, and cTNM stage were prognostic factors (all *P <*0.05), and the HR for each clinical variables can been found in **Supplement Table 1**. Furthermore, multivariate analyses found that only sex, T stage, N stage, and GTVp were independent prognostic factors (**Table 2**). Accordingly, a nomogram model was constructed based on such independent prognostic factors to depict their different weighted points (**Figure 1**). In addition, the 1-, 3-, and 5-year OS rates were predicted by the sum of these independent prognostic factor points. It can be easily seen that patients with higher scores were prone to have poorer clinical outcomes.

**Table 2:** Multivariable analysis of clinical variables to predict overall survival in the training cohort.

	HR	95% CI	P value
Gender	0.719	0.557-0.929	0.011
Clinical T stage	1.239	1.061-1.448	0.007
Clinical N stage	1.284	1.120-1.471	<0.001
GTVp	1.578	1.237-2.012	<0.001

HR, hazard ratio; CI, confidence interval.

**Figure 2:**
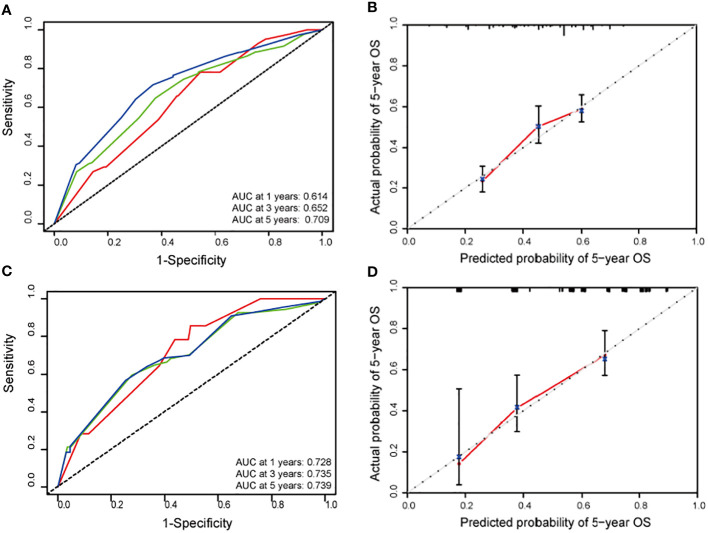
**(A)** Receiver operating characteristic (ROC) curves for 1-(red line), 3-(green line) and 5-year (blue line) overall survival (OS) according to the training cohort (TC) nomogram. **(B)** Calibration plot for predicting survival at 5-year in the TC nomogram. **(C)** ROC curves for 1-(red line), 3-(green line) and 5-year (blue line) OS according to the validation cohort (VC) nomogram. **(D)** Calibration plot for predicting survival at 5-year in the VC nomogram.

Next, the predictive function of the nomogram model was tested in the training cohort. Confirmed by 1,000 bootstrap resamples, the C-index for 5-year OS was 0.622 (95% CI: 0.59–0.654) (**Table 3**). The AUC value of the ROC for 5-year OS was 0.709 (95% CI: 0.661–0.758) (**Figure 2A**). Additionally, in the training cohort, the AUC value for 3-year overall survival was 0.700 (95% CI: 0.627–0.774) in the Surgery subgroup and 0.697 (95% CI: 0.627–0.767) in the CRT subgroup, indicating clinical relevance. Furthermore, the calibration curve for 5-year OS confirmed consistency between actual and predicted clinical outcomes (**Figure 2B**).

**Table 3:** The discriminatory ability of the nomogram model vs. AJCC stage.

	C-index (95% CI)	AUC (95% CI)	△C-index (*P* value)	NRI (*P* value)	IDI (*P* value)
TC Nomogram	0.622 (0.591-0.654)	0.709 (0.661-0.758)	–	–	–
TC AJCC stage	0.580 (0.548-0.612)	0.654 (0.605-0.703)	–	–	–
VC Nomogram	0.713 (0.656-0.771)	0.739 (0.655-0.823)	–	–	–
VC AJCC stage	0.659 (0.602-0.716)	0.689 (0.605-0.773)	–	–	–
TC Nomogram vs. AJCC stage	–	–	0.057 *P* <.001	26.6% *P* <.001	6.4% *P* <.001
VC Nomogram vs. AJCC stage	–	–	0.054 *P* = 0.020	23.9% *P* = 0.040	7.6% *P* <.001

TC, training cohort; VC, validation cohort; C-index, concordance index; CI, confidence interval; NRI, net reclassification index; IDI, integrated discrimination improvement.

NRI or IDI>0 indicate positive improvement, suggesting that the nomogram model achieved better prediction ability than AJCC stage. NRI or IDI<0 indicate diminished improvement, and the nomogram model’s prediction ability was less than that of the AJCC stage. NRI or IDI = 0 indicate that the nomogram model did not change.

Finally, this nomogram model was validated through an external independent cohort. For the validation cohort, the C-index of 5-year OS was 0.713 (95% CI: 0.656–0.771) (**Table 3**) and the AUC value of 5-year OS was 0.739 (95% CI: 0.655-0.823), which seemed better than those of training cohort (**Figure 2C**). The calibration curve demonstrates good agreement between actual and predicted OS (**Figure 2D**).

### 3.3 Comparison of the predictive accuracy between the nomogram and the AJCC staging system

In the comparison of nomogram model with the 8^th^ AJCC staging system, four indices including C-index, AUC, NRI, and IDI were compared (**Table 3**). For the training cohort, the C-indexes for the nomogram model and 8^th^ AJCC staging were 0.622 vs 0.580 (△C = 0.0570, 95% CI: 0.0271–0.08072, *P <*0.001) and 0.713 v. 0.659 (△C = 0.0541, 95% CI: –0.0026–0.0401, *P* = 0.020) for the validation cohort. Time-dependent ROC analyses showed that the AUC value of the nomogram model was significantly better than that of the 8^th^ AJCC staging for the training or validation cohort. With regard to the comparison of the NRI of 5-year survival between the nomogram model and 8^th^ AJCC staging, the discrimination ability of the nomogram model was increased by 26.6% and 23.9% in the two cohorts, respectively (all *P <*0.05). In addition, in the comparison of IDI of 5-year survival, that of the nomogram model increased by 6.4% and 7.6% in the training and validation cohorts, respectively.

**Figure 3:**
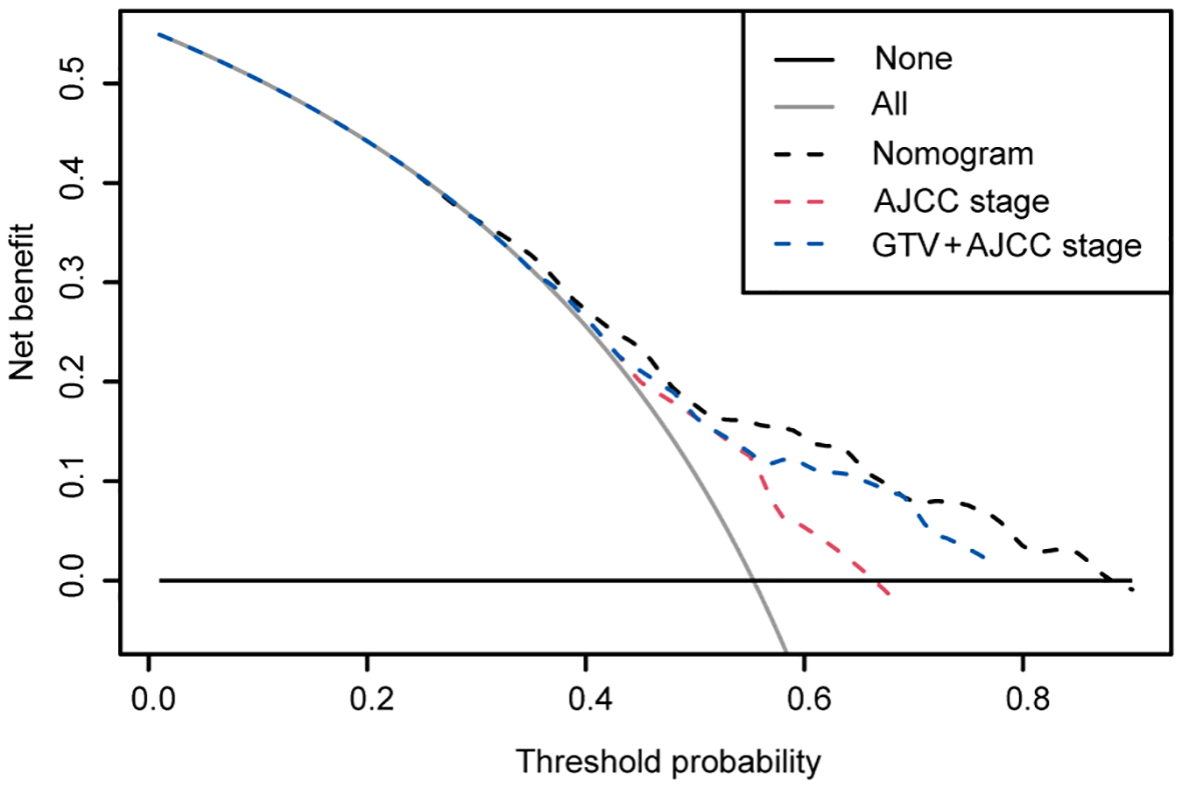
Decision curve analysis of the nomogram, the AJCC stage and GTV + AJCC stage in the training cohort. x-axis, threshold probability; y-axis, the standardized net benefit; gray line, the hypothesis that all patients survived for 5 years; black line, the hypothesis that all patients did not survive for 5 years. If one model achieves the highest net benefit compared with other models or any simple strategies at any given threshold, it is of clinical significance.

Moreover, the DCA confirmed our expectations. The 5-year DCA also suggested a better clinical benefit of the nomogram model compared with the 8th AJCC staging (**Figure 3**). In addition, it was also illustrated that GTVp was an excellent prognostic evaluation risk factor, and a combination of the GTVp and the 8th AJCC staging was markedly better than the 8th AJCC staging.

### 3.4 Risk-groups categorization

**Figure 4:**
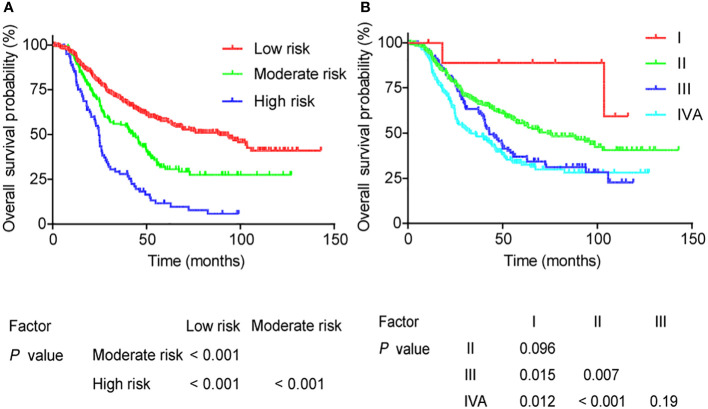
Overall survival for different groups as defined by **(A)** the nomogram model and **(B)** the AJCC stage.

Depending on the 5-year OS rate, risk scores were calculated to stratify patients into low, moderate, and high-risk groups in the training cohort. Statistically significant differences were ultimately found among these three subgroups (all *P <*0.05) (**Figure 4A**). Furthermore, 356, 132, and 80 patients were separately categorized into low, moderate, and high-risk groups, and their risk-score intervals were <152, 152–213, and >213, respectively. The 5-year OS rates for low, moderate, and high-risk groups were 86.1%, 54.5%, and 28.1%, respectively.

To further compare the nomogram with AJCC staging, the 5-year OS curves of AJCC staging are shown in **Figure 4B**. The 5-year OS rates gradually decreased as AJCC clinical staging increased as follows: 88.9%, 55.4%, 36.9%, and 32.5%. However, the OS curves of stages I and II were not well separated, and those of stages III and IVA stage did not significantly differ (all *P >*0.05).

## 4 Discussion

To the best of our knowledge, the present study is the first to develop a prognostic nomogram and risk stratification system for upper thoracic ESCC in a large cohort. In this study, a nomogram model was successfully constructed based on the independent prognostic factors of the training cohort, which achieved an accurate prediction of OS and exhibited good reproducibility, validated by an external cohort. Furthermore, this nomogram model was demonstrated to better predict OS and clinical survival benefit compared with the 8^th^ AJCC staging system. Last, the 5-year OS curves of low, moderate, and high-risk groups stratified by the nomogram revealed fairly good separation, whereas those of stages I and II were not well separated, and stages III and IVA did not significantly differ. These findings suggest that the nomogram model effectively predicted OS and provided risk stratification of upper thoracic ESCC.

In the present study, we first established a nomogram model on the basis of independent prognostic factors using multivariate analyses including sex, GTVp, cT stage, and cN stage. In general, discrimination and calibration are commonly used to evaluate the prognostic efficacy of a nomogram model (25). At present, the C-index is considered one of the most frequently employed measures to indicate the discrimination ability of a nomogram (25). However, as a rank-order statistic, the C-index considers that the survival state will vary with survival time, leading to systematic errors (12). Therefore, time-dependent ROC analyses were applied to address the discrimination deficiency of the C-index (26).

Furthermore, calibration of the nomogram was assessed by the calibration curve, representing the agreement between the actual and predicted clinical outcomes (27). Accordingly, the C-index and AUC value for 5-year OS were 0.622 and 0.709, respectively, which showed favorable discrimination and calibration ability to predict OS. Finally, the constructed nomogram was successfully validated by an external independent cohort with a C-index of 0.713 and an AUC value of 0.739, which confirmed the prognostic efficacy and reproducibility of the nomogram model.

To further demonstrate the applicability of this nomogram model, it must be compared with the 8^th^ AJCC staging system for upper ESCC. We first found that the C-index and AUC value of the nomogram of the training or validation cohort were markedly better than those of the 8^th^ AJCC staging. Furthermore, novel indices, including NRI and IDI, were employed as well to investigate the discrimination ability of the nomogram. NRI and IDI were originally proposed to characterize the improvement in accuracy for predicting patients’ outcomes (15). In the calculation of the NRI and IDI, the improvements in sensitivity and specificity are summed, which better evaluate the effectiveness of the model (25). The present study shows that the NRI and IDI of the nomogram significantly increased as compared to those of the 8^th^ AJCC staging, which demonstrates the improvement in the former of the predictive performance of OS.

Moreover, DCA analyses exhibited a higher net benefit in the nomogram compared with the AJCC staging at any given threshold, representing better clinical usefulness of the nomogram. The DCA is one of decision-analytical measures that plot the net benefit achieved by making decisions based on the prognostic model (23). However, the traditional prognostic model is based upon the AJCC staging system, which may achieve less clinical benefit than the nomogram involving other independent prognostic factors. In the present study, GTVp and sex served as additional independent factors included in the nomogram, which conferred better clinical benefit. In addition, GTVp combined with the AJCC staging possessed significantly better clinical benefit than the AJCC staging, which suggested that GTVp can aid to improve the predictive ability of clinical stage and should be added to the TNM staging system in the future. Similarly, body mass index, absolute lymphocyte counts, neutrophil-to-lymphocyte ratio, and wall thickness were incorporated into the nomogram for patients with ESCC receiving definitive chemoradiotherapy, which also demonstrates superior clinical usefulness compared with AJCC staging (9).

Finally, a risk stratification system according to the nomogram model was developed to distinguish patients with different mortality risk levels. When comparing the risk stratification system with the AJCC staging system, the 5-year OS curves among low, moderate, and high-risk groups showed fairly good separation, whereas those among clinical stage could not be well separated, particularly for stages III and IV. Thus, the risk stratification system was better for predicting OS than AJCC staging, which appeared to be more precise, to guide treatment. For the high-risk group with upper ESCC, the present radical treatment seems inadequate, and more aggressive treatment strategies are urgently required to improve prognosis. Recently, the administration of an additional targeting agent during radiotherapy for older patients or neoadjuvant chemoradiotherapy for locally advanced EC, such as nimotuzumab, were initially reported as a feasible anticancer strategy (28, 29). Therefore, further studies are warranted to confirm such targeting and neoadjuvant therapeutic effects on upper thoracic ESCC. Furthermore, the addition of immunotherapy for EC, such as immune checkpoint inhibitors, demonstrates its clinical benefit (30). For the low-risk group, certain aggressive treatments, including neoadjuvant or adjuvant chemotherapy, may be unnecessary, which may effectively decrease the expenses of oncotherapy and the adverse effects of chemotherapy.

Nevertheless, we acknowledge two limitations in our study. First, the study was conducted through retrospective analysis, regardless of the large cohort, and thus treatment-selection bias may be unavoidable. A prospective, multicentral, and randomized study is needed to verify the prognostic precision of the nomogram model for upper thoracic ESCC. Second, this analysis did not incorporate other potential prognostic factors such as hematology indicators and pathological characteristics, which may further improve the prognostic accuracy of the nomogram model and refine risk stratification (31). Hence, a nomogram that is composed of more comprehensive prognostic factors is required as well.

## 5 Conclusion

In conclusion, a nomogram was successfully modeled to effectively predict OS of patients with upper thoracic ESCC, which may provide a clinical reference for precise prediction of OS and further stratification of the management of patients at risk.

## Data availability statement

The raw data supporting the conclusions of this article will be made available by the authors, without undue reservation.

## Ethics statement

The studies involving human participants were reviewed and approved by the Institutional Review Board of Fujian Medical University Cancer Hospital (No. SQ2020-063-01). The patients/participants provided their written informed consent to participate in this study.

## Author contributions

YX and QW participated in the design of the study, YL and BZ performed the experiments and the statistical analysis, and drafted the manuscript. BZ and QH performed the experiments and the statistical analysis, YL and BZ drafted the manuscript and assisted with the manuscript preparation. YYe, YC, BC and MY collected the data. YYa and JC examined and revised the manuscript. All authors contributed to the article and approved the submitted version.
