# Supplementary material for: Development of a prognostic nomogram and risk stratification system for upper thoracic esophageal squamous cell carcinoma
Source: Front Oncol. 2023 Apr 12;13:1059539. doi: 10.3389/fonc.2023.1059539 (PMC10130360; doi:10.3389/fonc.2023.1059539)
Supplement: Supplementary file 1 [file Table_1.pdf]

Supplement Table 1 Univariate analysis of clinical variables to predict overall survival in the training cohort

	HR	95% CI	*P* value
Gender			
Male	Ref		
Female	0.636	0.494-0.820	<0.001
Age (years)			
< 60	Ref		
≥ 60	1.041	0.835-1.297	0.721
LNM			
No	Ref		
Yes	2.042	1.615-2.581	<0.001
Tumor length (cm)			
≤ 5	Ref		
> 5	1.471	1.177-1.839	0.001
GTVp (cm3)			
< 30	Ref		
≥ 30	2.036	1.625-2.550	<0.001
Clinical T stage			
T1	Ref		
T2	1.162	0.459-2.937	0.752
T3	2.058	0.844-5.020	0.113
T4	2.684	1.097-6.569	0.031
Clinical N stage			
N0	Ref		
N1	1.597	1.238-2.060	<0.001
N2	2.138	1.592-2.871	<0.001
N3	3.560	1.866-6.792	<0.001
8th AJCC stage			
I	Ref		
Ⅱ	3.039	0.751-12.298	0.119
III	4.468	1.096-18.214	0.037
Ⅳ	5.425	1.340-21.970	0.018
Treatment			
Surgery	Ref		
CRT	1.393	1.089-1.781	0.008
Surgery+CRT	1.038	0.760-1.416	0.815

Abbreviation: HR, hazard ratio; CI, confidence interval.
